# Safety and efficacy of a side-to-side duodeno-ileal anastomosis for weight loss and type-2 diabetes: duodenal bipartition, a novel metabolic surgery procedure

**DOI:** 10.1186/s13022-015-0015-0

**Published:** 2015-10-14

**Authors:** Michel Gagner

**Affiliations:** Department of Surgery, Herbert Wertheim School of Medicine, Hopital du Sacre Coeur, Florida International University, 315 Place D’Youville, Suite 191, Montreal, QC H2Y 0A4 Canada

**Keywords:** Duodeno-ileal anastomosis, Duodeno-jejunal bypass, Bariatric surgery, New technology, Weight loss surgery, Obesity treatment, Anastomotic device, Duodenal bipartition

## Abstract

**Background:**

Partial bypass of the GI tract may promote weight loss by decreased absorption of nutrients and changes in incretins. The aim of the study was to evaluate the safety and efficacy of performing a side-to-side duodeno-ileal anastomosis.

**Methods:**

Seven 40–50 kg female Yorkshire pigs were allocated to a duodeno-ileal anastomosis (DIA), and were compared to a control group (SHAM). Swine’s weights were followed for 56 days. Gastroscopies were also performed at 28 days. Blood samples were also taken at regular intervals (CBC and Basic biochemistry profiles). At autopsy, gross changes and histological changes of the liver, duodenum and ileum samples were performed.

**Results:**

While the SHAM group gained 33.2 % more weight at 56 days, the DIA group had shown a weight loss of −6.8 %, for a difference of 40.0 % between the 2 groups (p < 0.05). One pig developed an incisional hernia. Gastroscopies demonstrated normal healing without ulceration or inflammation at 28 days. Histological examination of the anastomosis at 56 days showed normal and smooth healing, with absence of liver toxicity.

**Conclusion:**

In this porcine model with short follow-up, a side-to-side duodeno-ileal anastomosis provided excellent weight loss without apparent nutritional or grossly aberrant histological changes.

## Background

In the purest form of malabsorptive surgery for weight loss, the jejunoileal bypass (JIB), one of the earliest types of bariatric surgery, was introduced with its many variations, four and five decades ago. The JIB was performed end-to-side, with the proximal thirty centimeters jejunum anastomosed to the distal 15 cm of ileum, or end-to-end, with bypassed small bowel derived end-to-side to the colon. In both instances more than 90 % of small intestine was bypassed, unexcised, excluding it from the alimentary channel leaving a blind end, causing bacterial overgrowth.

Excellent weight loss and complete resolution of type-2 diabetes mellitus were reported after JIB [1, 2]. However, a variety of complications related to JIB were reported, including: hypoalbuminemia, hypokalemia, hypocalcemia, hyperbilirubinemia, migratory polyarthralgias, calcium oxalate urinary calculi, and elevated liver enzymes levels and deaths due to liver failure [3, 4]. Diarrhea and flatulence were common. The excluded intestinal segment was associated with various problems, including intussusceptions; bypass enteritis, and colonic pseudo-obstruction. Other authors reported that the risk of progressive liver disease existed indefinitely and that ongoing careful follow-up was necessary [5, 6].

**Fig. 1:**
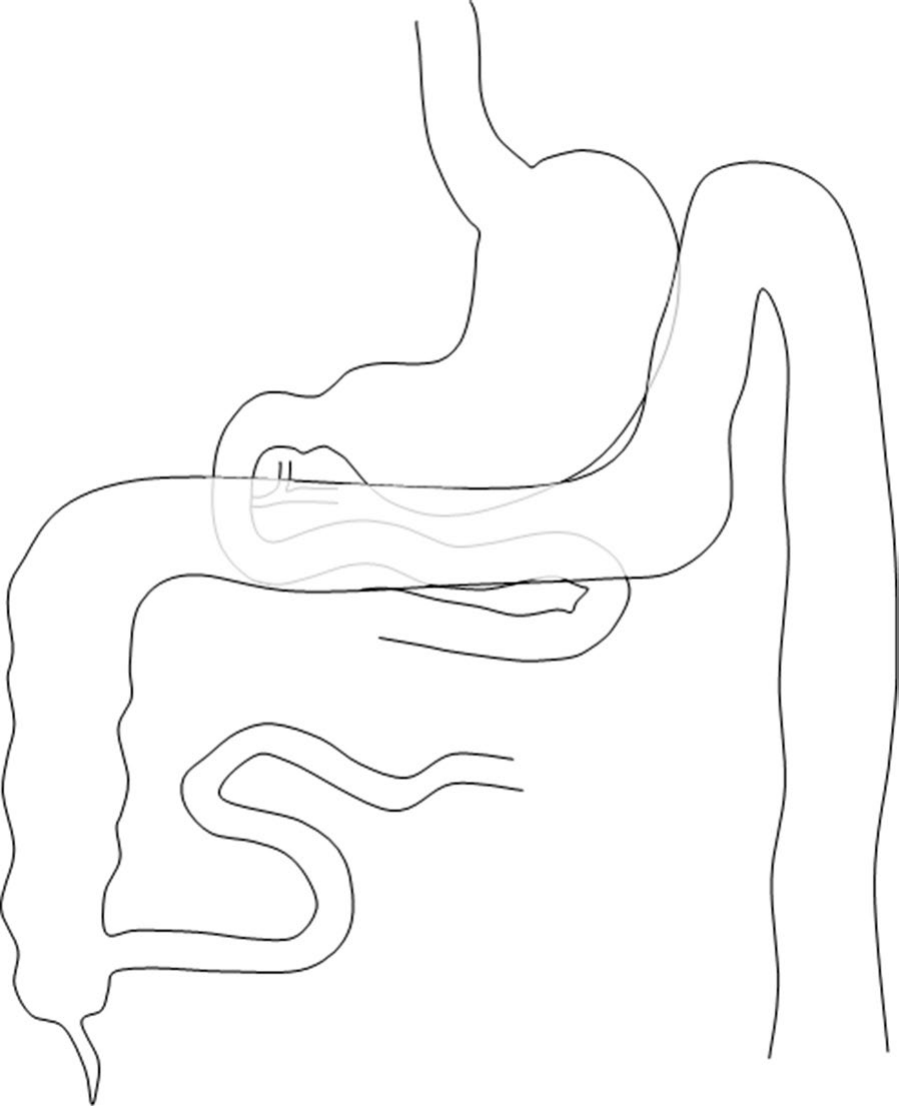
Position of the right transverse colon overlying the 3rd and fourth portions of the duodenum, with distal ileum lying in close proximity

**Fig. 2:**
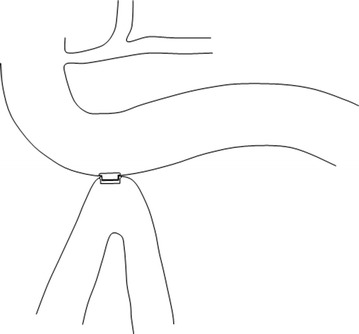
Endotract TM device implanted between the lower side of the 3rd portion of the duodenum and upper side of distal ileum, by open enterotomies

**Fig. 3:**
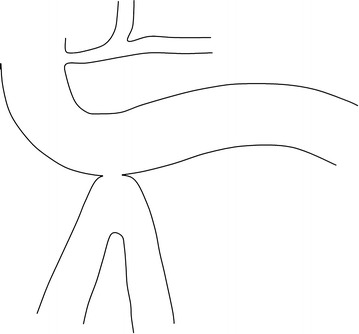
After 7–10 days of tissue compression, the Endotract TM device has passed with bowel transit, leaving a permanent side-to-side duodeno-ileal anastomosis

**Fig. 4:**
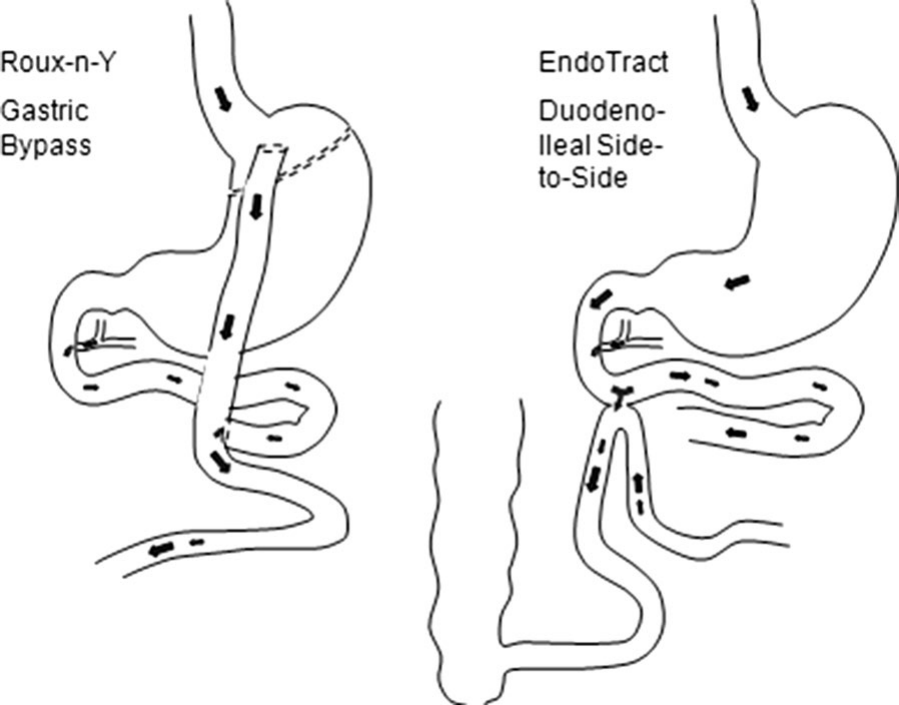
Comparison of digestive flow between a gastric bypass and a side-to-side duodeno-ileal anastomosis. Alimentary flow and biliopancreatic secretions are mixing in a Y channel (100–150 cm in mid jejunum) in a gastric bypass, while in a side-to-side duodeno-ileal anastomosis, the alimentary flow is divided between a regular jejuno-ileal channel and a distal ileal channel, and biliopancreatic secretions are mixing in the proximal duodenum

However, when a 90 % small bowel resection in germ free rats is compared to a 90 % small bowel bypass, the resected animals have remained with normal liver histology after a prolonged period. This means that any blind limb is possibly responsible for liver insufficiency. Therefore the development of a new surgical malabsorptive procedure should not involve any blind segment [7]. A model of partial malabsorptive bypass is constructed with a side-to-side anastomosis between the 3rd portion of the duodenum and last 50 cm of the ileum (anatomically in close proximity) Figs. 1, 2, 3, allowing a partial flow of nutrients to move in the proximal jejunum for normal mineral absorption and caloric intake, while a portion is bypassed into the distal ileum, causing a decreased absorption resulting in weight loss. Since both limbs have flow, bacterial overgrowth is of a lesser concern theoretically comparable to Roux-en-Y gastric bypass (Fig. 4). To perform this anastomosis, a compression anastomotic device was used for its simplicity.

Compression anastomotic devices for the performance of gastrointestinal anastomosis have been available for more than a century and used extensively in colon surgery in its resorbable form, and more recently simulating a commercial end-to-end anastomotic device [8–10].

The aim of the study was to evaluate the safety and efficacy of using a compression anastomotic device to create a permanent anastomosis between the small bowel and duodenum. Also, to evaluate the effect of a side-to-side duodeno-ileal anastomosis on weight loss, in the short term.

## Methods

The animal protocol was approved by the institutional Animal care and use Committee (IACUC) of American Preclinical Services, LLC (APS) facility licensed with the United States Department of Agriculture. We used 7 Yorkshire pigs, >2 months old, weighing approximately 40–60 kg, housed individually. The porcine diet consisted of a fixed formula certified by the manufacturer to be free of environmental contaminants; tap water was given ad libitum.

Blood samples were taken for minimal hematology parameters (red blood cell count, hemoglobin, hematocrit, platelet count, white blood cell count and differential), minimal serum biochemistry parameters (Urea nitrogen (BUN), creatinin, total protein, albumin, aspartate aminotransferase (AST), gamma glutamyltransferase (GGT), glucose, sodium, potassium, chloride, calcium, phosphorus, bicarbonate). Prior to surgery, animals have been administered a 3 days bowel prep with 2 l/day of Golitely (PEG-3350, Braintree Laboratories, Inc., Braintree, MA, USA) and Ensure (Abbott Nutrition, Columbus, OH, USA) to cleanse the colon, and were fasted the night before except for water. Preoperative medication included; Telazol 2–8 mg/kg for anesthesia induction (Tiletamine HCl and Zolazepram HCl, Animal Healthcare, Wyeth (now Pfizer, Inc.), Fort Dodge, IA), Xylazine 2–8 mg/kg (Bayer Healthcare, Leverkusen, Germany) for anesthesia induction, Buprenorphine 0.01–0.05 mg/kg for pain management (Buprenex, Reckitt & Colman Pharmaceuticals, Inc., Richmond, VA, USA), and Oxytetracycline (long acting) 20 mg/kg (Hebei New Century Pharmaceutical Co., Ltd., Hebei, China)for infection prophylaxis.

Four animals were allocated to a side-to-side duodeno-ileostomy with the compression anastomotic device and 3 to a control group. After endotracheal intubation, anesthesia was maintained with isoflurane in 100 % O_2_ and propofol at 2–8 mg/kg, with an intravenous Ringers’ lactate solution at 2–10 ml/kg/h. After laparotomy with a 25 cm upper midline incision, a duodenotomy of approximately 2.5 cm was created anterior to admit the proximal part of the compression anastomotic device, and an ileotomy approximately 50 cm from the ileocecal valve was made to insert the distal part compression anastomotic device. The anastomosis was performed by compression of both parts away from the duodenotomy and ileotomy (Fig. 2). Both openings of the small bowel were closed with a running suture of 3-0 Vicryl (Polyglactin-910, Ethicon, Cincinnati, OH). The control group had both enterotomies closed with a running suture only. A liver biopsy was also performed by a wedge. The abdominal wound is closed with Vicryl 1-0 for fascia and 3-0 for skin.

During recovery in the pen and postoperative period, the animals received Buprenorphine 0.01–0.05 mg/kg IM as needed, ketoprofen 1.8–2.2 mg/kg IM daily for the first 3 days (Ketofen, Fort Dodge Animal Health, Fort Dodge, IA, USA), Prilosec 20 mg once daily (Omeprazole, AstraZeneca, Wilmington, DE, USA), and Oxytetracycline (long acting) 18–25 mg/kg IM on day 3. During the first 24 h, the animals were allowed to drink water, and afterwards soft food was introduced to gradually progress to a normal solid diet over 10 days. Elimination of the device was recorded, including signs of infection. Blood samples were taken at day 0, 3 and 56.

On Day 28, a gastroscopy (Olympus, GIF-2T20, 11.2 mm diameter) was performed under general anesthesia, using a similar protocol, and photographs were obtained of the anastomosis, and nearby intraluminal organs, to assess patency, diameter, and degree of inflammation and presence of macroscopic abnormalities. An attempt was made to measure the intestinal shunting from the procedure by introducing approximately 25 radiopaque doughnut-type markers (Sitzmark, Konsyl Pharmaceutis, Inc., TX, USA) in the proximal stomach for a gastrointestinal transit study, by taking abdominal x-rays every 2 h for 6 h.

At 8 weeks, euthanasia and necropsy of the abdominal cavity is performed. Samples of the liver were taken at the time of the anastomotic procedure (pre-sample) and at necropsy (left medial liver lobe, post-sample) and immersion-fixed in 10 % neutral buffered formalin (NBF). The gastrointestinal tract was rinsed with water to remove food content and images were taken of each excised anastomotic site. Additionally, a sample of the right gluteus maximus was procured from each animal. All tissue samples were immersion-fixed in 10 % NBF. Two sections from each anastomotic site were trimmed, sections of pre- and post-anastomosis lever samples, and a section of right gluteus maximus skeletal muscle were taken for histological processing. The sections were placed in labeled cassettes and tissues were processed through a graded series of alcohols, embedded in paraffin, cut with a rotary microtome to approximately 5 µm in thickness, mounted on microscopic slides, and stained with hematoxylin and eosin (H & E) and Masson’s trichrome stains. American Preclinical Services (APS) sent the digital images taken at necropsy, completed gross pathology forms, trim sheets, and microscopic slides to a board-certified veterinary pathologist for independent interpretation. The sections of the anastomotic sites were evaluated for healing response and the presence of inflammation, infection, or dehiscence at the site of apposition.

## Results

**Fig. 5:**
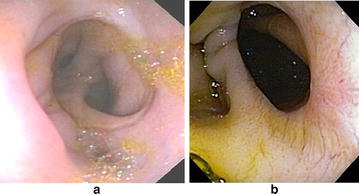
**a** Duodenoscopy of the 3rd portion of the duodenum, showing a healed side-to-side duodeno-ileal anastomosis, with proximal ileum on the *right* and distal ileum on the *left* of the figure. **b** Duodenoscopy of the 3rd portion of the duodenum from a different animal, showing a healed side-to-side duodeno-ileal anastomosis, with distal duodenum on the far *right* of the figure

At 28 days post surgery all pigs are healthy, with good appetite, eating the proposed diet with normal feces. However, one pig had developed a small, external incisional hernia, which had to be corrected. At 28 days duodenoscopy of all animals showed a widely patent healed side-to-side duodeno-ileal anastomosis, with proximal ileum on the right and distal ileum on the left (Fig. 5a, b). The gastroscope was able to pass through the anastomosis, in all limbs. There was no evidence of gross ulcerations in all parts of the duodenum, nor in the ileum. There was no visible inflammation either. The anastomosis itself revealed a smooth transition between both mucosae. Since the pigs had been fasting and mostly bilious fluids with saponification from the air insufflations was visible. We were not successful in determining various gastrointestinal transit times from both limbs, as the transit of markers were too slow (the majority remained in the stomach during the study period).

**Fig. 6:**
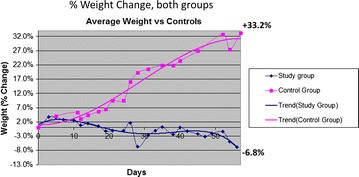
Graph of % of weight change in animals that had a side-to-side duodeno-ileal anastomosis (study group) versus sham controls, over time in days. At 56 days, control animals had gained 33.2 % of weight, while study animals had lost 6.8 % of weight

Weight progression in both the DI animals and controls were recorded weekly and plotted for comparison. In fact for better understanding of the progression, the mean percentage of weight change from baseline in animals that had a side-to-side duodeno-ileal anastomosis (study group) versus sham controls, over time in days was projected (Fig. 6). At 56 days, control animals had gained 33.2 % of weight, while study animals had lost 6.8 % of weight.

All Blood tests are reported in Table 1 and 2. Mean values for hematological profiles at baseline, day 3 and 56 shows a decline in RBC count (−21 %), hemoglobin (−15 %), hematocrit (−19 %) and increase in platelet counts (+60 %) and WBC counts (+16 %), at 56 days compared to baseline. Equally, mean values of serum biochemical profiles at baseline, day 3 and 36 are presented in Table 2. After a slight increase (+33 %) in serum glucose at day 3, presumably from stress response after surgery, the mean value returns within normal range at 56 days. There is a notable decrease in serum total protein and albumin at 56 days by 23 and 25 %, attributable to a fixed diet. Equally, the BUN has increased by threefold at 56 days. Nitrogen loss maybe attributable to inadequate intake of calories from a restricted diet and decreased absorption from bypass of the GI tract. A slight and subtle change of serum calcium (−11 %) and phosphorus (−17 %) is observed at 56 days, but within normal range, and may parallel the decrease in serum proteins. The serum potassium, sodium, chloride and bicarbonate remained within normal range. The only serum liver enzyme measured remained constantly normal.

**Table 1:** Mean values of hematological profiles at baseline, day 3 and 56

	Time after duodeno-ilial anastomosis		
	Baseline	Day 3	Day 56
RBC count	6.46	6.92	5.09
Hemoglobin (g/DL)	11.08	12.05	9.40
Hematocrit (%)	34.65	36.48	28.10
Platelet count	280.25	305.50	448.50
WBC count	14.55	16.83	16.85

*WBC* white blood count

**Table 2:** Mean values of serum biochemical profiles at baseline, day 3 and 56

	Time after duodenum-ileal anastomosis		
	Baseline	Day 3	Day 56
Glucose	75.75	101.25	88.50
AST	35.25	29.00	39.25
Total protein	6.53	6.40	5.05
Albumin	3.45	3.30	2.58
Urea N	5.00	8.75	15.00
Creatinine	1.38	1.30	1.15
Phosphorous	7.20	7.00	6.00
Calcium	10.23	9.60	9.10
Sodium	141.75	140.25	136.25
Potassium	3.65	3.80	4.25
Chloride	102.00	98.00	103.25
Bicarbonate	27.75	29.00	26.75
Gamma-GT	26.75	24.25	21.25

*AST* alamine serum trasnferase, *N* nitrogen, *GT* glutamine transferase

**Fig. 7:**
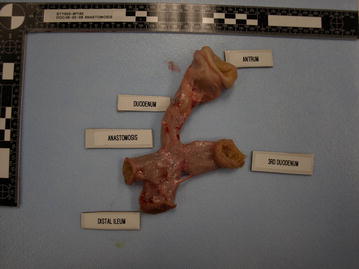
Macroscopic external view of a side-to-side duodeno-ileal anastomosis at 56 days

**Fig. 8:**
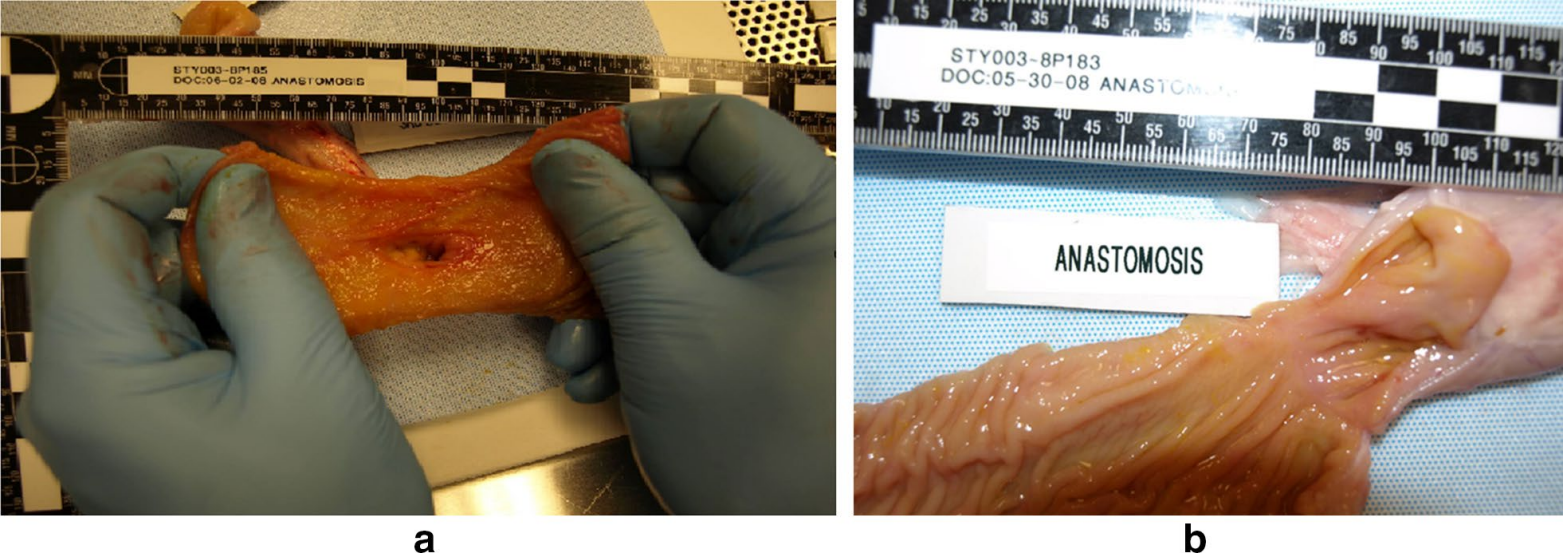
**a** Macroscopic luminal view (from the ileum side) of a side-to-side duodeno-ileal anastomosis at 56 days. **b** Macroscopic luminal view of a longitudinal opening of a side-to-side duodeno-ileal anastomosis at 56 days

At necropsy, general flimsy adhesions were encountered near the anastomosis; the liver had a normal macroscopic appearance. Each gastroduodenal areas were harvested for measurements and histological sampling. Macroscopic external view of a side-to-side duodeno-ileal anastomosis revealed a smooth surface serosal appositions (Fig. 7), and inside the anastomosis could admit the index finger (Fig. 8a), and once this duodenoileal anastomosis was opened along its longitudinal axis, a smooth surface was revealed (Fig. 8b).

**Fig. 9:**
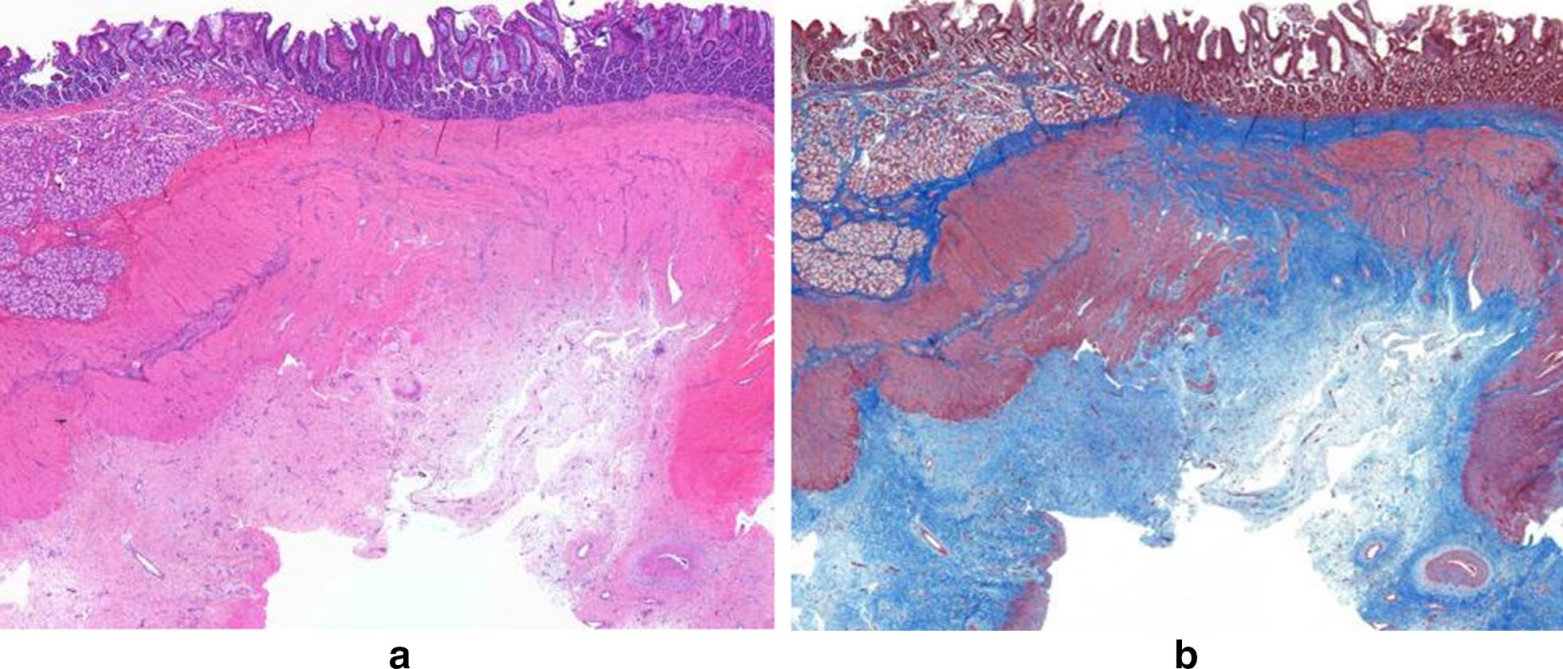
**a**, **b** Longitudinal section through the well healed duodenal-ileal anastomotic site. Note the apposition of all intestinal layers. The duodenal mucosa is contiguous with the ileal mucosa. Fibrous connective tissue (*blue* in trichrome stain) separates the muscle layers of the two portions of the intestine. The serosa appears slightly edematous. The lymphatics in the ileal submucosa appear dilated. There is no evidence of infection, inflammation or dehiscence at the anastomotic site. **a** H & E stain; **b** Masson’s trichrome stain. Both images ×20 magnification

**Fig. 10:**
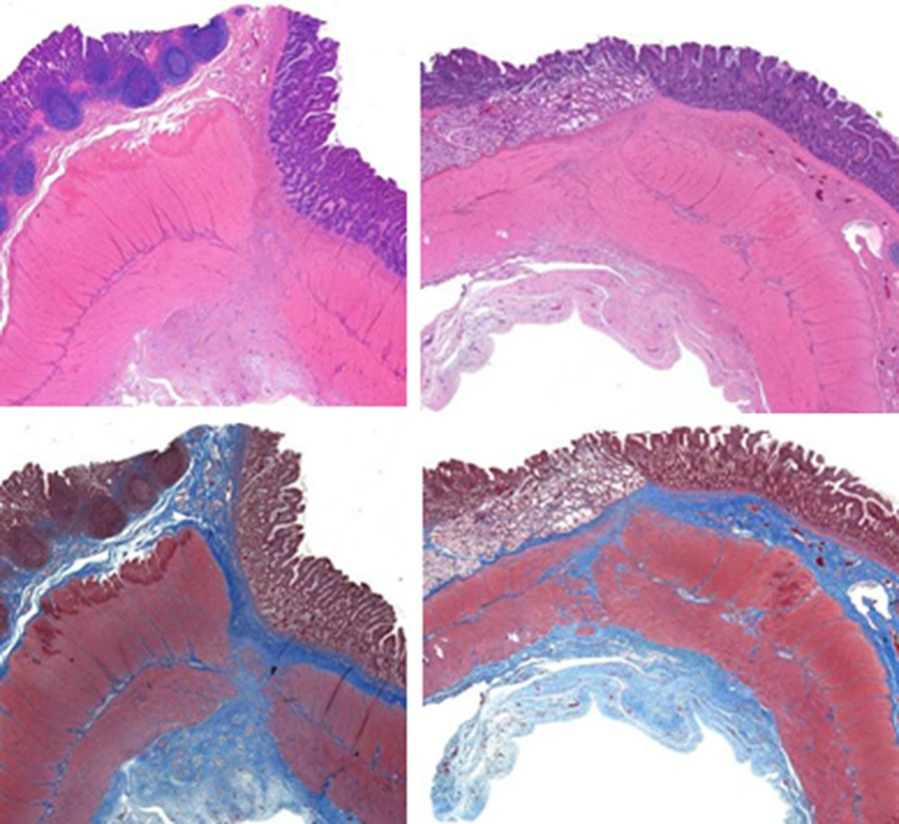
**a**–**d** Longitudinal sections through the well healed duodenal-ileal anastomotic site. It appears that all layers of the intestine are apposed (muscular layers not closely apposed) although only a small portion of the ileal mucosa is present in the first second (**a**, **b**) and the mucosa overlying the anastomotic site is absent in the second section (**c**, **d**). Abundant fibrous connective tissue (*blue* in trichrome stain) separates the muscle layers of the two portions of the intestine. The serosa appears slightly edematous and serosal vessels appear prominent with perivascular edema. There is no evidence of infection, inflammation or dehiscence at the anastomotic site. All images—×20 magnification. **a**, **c** H & E stain; **b**, **d** Masson’s trichrome stain

**Fig. 11:**
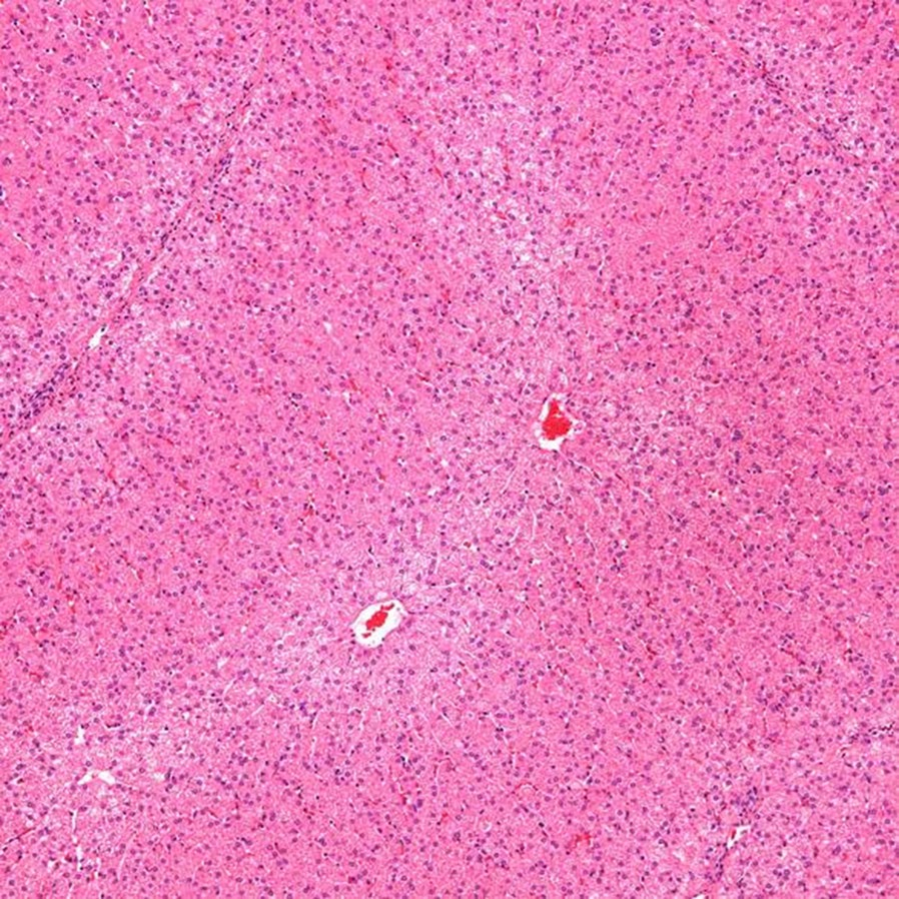
Liver histology at 56 days. Subtle centrilobular hepatocellular swelling and granularity (consistent with glycogen deposition) is noted in this animal. H & E stain, ×100 magnification

All layers of the intestine were well healed with good apposition of the mucosa and muscular layers of the duodenum and ileum. A variable amount of fibrous connective tissue was noted in the muscular layers of the two apposed edges of the intestine and extended into the surrounding muscle bundles. The muscle layers at the anastomotic site appeared to align (Figs 9, 10). The vasculature throughout the intestinal sections appeared normal with no evidence of thrombus formation or occlusion. Mild serosal edema was seen and serosa vessels appeared prominent with some perivascular edema noted. No evidence of infection, inflammation, or dehiscence was noted at any of the anastomotic sites. Two (2) of the four (4) post-liver samples taken were considered within normal limits (WNL) and the other two (2) of four (4) samples showed subtle changes of hepatocellular swelling with glycogen accumulation (Fig. 11). Similar microscopic changes of glycogen accumulation can also be seen during various stages of fasting in animals. No evidence of muscle fiber vacuolization, loss, inflammation, fatty infiltration or increased fibrous connective tissue deposition was seen in any of muscle sections examined.

## Discussion

The use of a new compressive device for GI anastomosis allowed a safe and effective creation of an anastomosis between two portions of the small bowel. The anastomosis created was robust, healthy and permanent, which facilitated a partial diversion of nutrient flow and thus altered nutrients absorption, causing effective weight loss in this porcine model with short follow-up. A side-to-side duodeno-ileal anastomosis provided excellent weight loss, without diarrhea or grossly aberrant histological changes, especially in the liver. However, a notable decline in serum total protein and albumin levels (and elevated BUN) may point towards inadequate protein/calorie absorption. In the absence of proper nitrogen balance measurements, resting energy expenditure, one cannot conclude that inadequate intake resulted in this early phenomenon, especially taking into account the energetic and protein needs to heal a midline laparotomy and 2 enterotomies. It is also possible that if the animal had access to an ad libitum diet that serum protein and albumin levels would have been maintained.

In the real clinical world, humans have free access to nutrients, and are provided with protein supplementation and nutritional counseling after surgery. It is expected that any malabsorptive procedure, must include these components and serial serum levels of protein, albumin, minerals, fat soluble vitamins and liver enzymes, similarly to gastric bypass, biliopancreatic diversion with or without duodenal switch must be carried out at regular intervals. Equally remarkable, is that the bypassed intestine in the pig, is greater with (97 % bypass, 50 cm from 18 meters) a ratio of 1: 36 when compared to humans (90 % bypass, 50 cm from 5 meters) with a ratio of 1:10 [11]. Therefore, this phenomenon may be seen less in humans.

Recent literature still appears on jejuno-ileal bypass and its’ modifications. Recently, Fazel et al. have reported a successful consecutive series of forty-three patients who underwent a modified jejunoileal bypass where the defunctionalyzed limb was anastomosed to the gall bladder and cecum, resulting in a loss of 43 kg (or 15 kg/m^2^ of BMI) at 5 years, without changes in liver histology [12].

One of the main reason why jejunoileal bypass was abandoned were reports of deaths from liver failure. Meinhardt and colleagues have followed carefully 50 consecutive patients who underwent JIB, in which liver biopsies were performed intraoperatively in 41 patients and in follow-up of 31 patients. With good weight loss at a mean of 67 months, no deaths occurred from liver failures and liver histology was stable [5].

The team of Rosina on 49 patients extensively studied bacterial overgrowth. Only 45 % of patients had some colonic micro flora in the excluded limb of jejunoileal bypass. The colonization appeared to correlate with clinical symptoms of bloating, migratory arthralgia, and rashes and skin lesions. But conversely, the positive cultures were not always associated with symptoms. No specific bacteriology was associated with this phenomenon. According to Rosina, the “success of an intestinal bypass may depend not only on anatomic and functional adaptation to the new, surgically created conditions, but also to the attainment of microbiological equilibrium in the intestinal ecosystem” [13].

Riordan et al. have reported that bacterial overgrowth does not necessarily correlates with neither liver damage nor increased intestinal permeability in human subjects [14].

The main advantage of a duodeno-ileostomy would be the fast ileal stimulation, causing an early incretin release and offering a potential tool for the resolution of type-2 diabetes. Recent hypothesis concerning the resolution of type-2 diabetes after weight loss surgery seems to point out that distal bowel stimulation may promote the production of glucagon-like peptide-1 (GLP-1) from the ileal and colonic L cells. There has been some evidence of this phenomenon when ileal transposition has been performed in Goto-Kakizaki type-2 diabetic rats [15]. Mason had proposed an ileal transposition to promote the early release of GLP-1 for the cure of type-2 diabetes [16]. Although we did not measure this hormone in pigs after duodeno-ileostomy, we postulate that an early release of GLP-1 will be a main endocrine feature of this operation.

Peptide YY (PYY) is also released from the distal small bowel endocrine cells is released in the circulation after a fatty meal, and PYY seemed to appear in the ileal lumen at greater concentration when glucose is used predominantly in the diet [17]. In fact when oleic acid is infused into the duodenum, PYY is released approximately 10–30 min after. The site of production of circulating PYY appears to be the ileum, colon and rectum. If an ileocolectomy is performed, an abolished production of PYY to intraduodenal stimulation of oleic acid is observed. This release is not mediated by neural pathway, but solely from endocrine nature [18].

In turn, the increasing concentration of intravenous infusion of PYY reduces the glucose stimulated insulin release. This suggests that PYY affects the Beta-cell function by a possible autonomic regulation [19]. Similarly, we are postulating that an early ileal release of PYY will occur after a side-to-side duodeno-ileostomy, and could be one hypothesis behind the effective weight loss seen in these animals.

## Conclusion

In this porcine model with short follow-up, a side-to-side duodeno-ileal anastomosis provided excellent weight loss without apparent nutritional or grossly aberrant histological changes. This intervention is likely to cause weight loss by numerous mechanisms including decreased food absorption and decreased satiety from endocrine stimulation [20, 21].
